# Supplementary material for: Infection- or vaccine mediated immunity reduces SARS-CoV-2 transmission, but increases competitiveness of Omicron in hamsters
Source: bioRxiv. 2022 Jul 29:2022.07.29.502072. Preprint. [Version 1] doi: 10.1101/2022.07.29.502072 (PMC9387121; doi:10.1101/2022.07.29.502072)
Supplement: Supplement 1 [file media-1.pdf]

Infection- or vaccine mediated immunity reduces SARS-CoV-2 transmission, but increases competitiveness of Omicron in hamsters

Julia R. Port*[1], Claude Kwe Yinda*[1], Jade C. Riopelle[1], Zachary A. Weishampel[1], Taylor A. Saturday[1], Victoria A. Avanzato[1], Jonathan E. Schulz[1], Myndi G. Holbrook[1], Kent Barbian[2], Rose Perry-Gottschalk[3], Elaine Haddock[1], Craig Martens[2], Carl. I. Shaia[4], Teresa Lambe[5$], Sarah C. Gilbert[5], Neeltje van Doremalen#[1], Vincent J. Munster#[§1]

1. *Laboratory of Virology, Division of Intramural Research, National Institute of Allergy and Infectious Diseases, National Institutes of Health, Hamilton, MT, USA*
2. *Genomics Research Section, Research Technologies Branch, Division of Intramural Research, National Institute of Allergy and Infectious Diseases, National Institutes of Health, Hamilton, MT, USA*
3. *Rocky Mountain Visual and Medical Arts Unit, Research Technologies Branch, Division of Intramural Research, National Institute of Allergy and Infectious Diseases, National Institutes of Health, Hamilton, MT, USA*
4. *Rocky Mountain Veterinary Branch, Division of Intramural Research, National Institute of Allergy and Infectious Diseases, National Institutes of Health, Hamilton, MT, USA*
5. *The Jenner Institute, Nuffield Department of Medicine, University of Oxford, Oxford, UK*

* These first authors contributed equally

# These senior authors contributed equally

[§] Corresponding author. vincent.munster@nih.gov

[$] New address: Chinese Academy of Medical Science Oxford Institute; Oxford Vaccine Group, Department of Paediatrics, University of Oxford, Oxford, UK

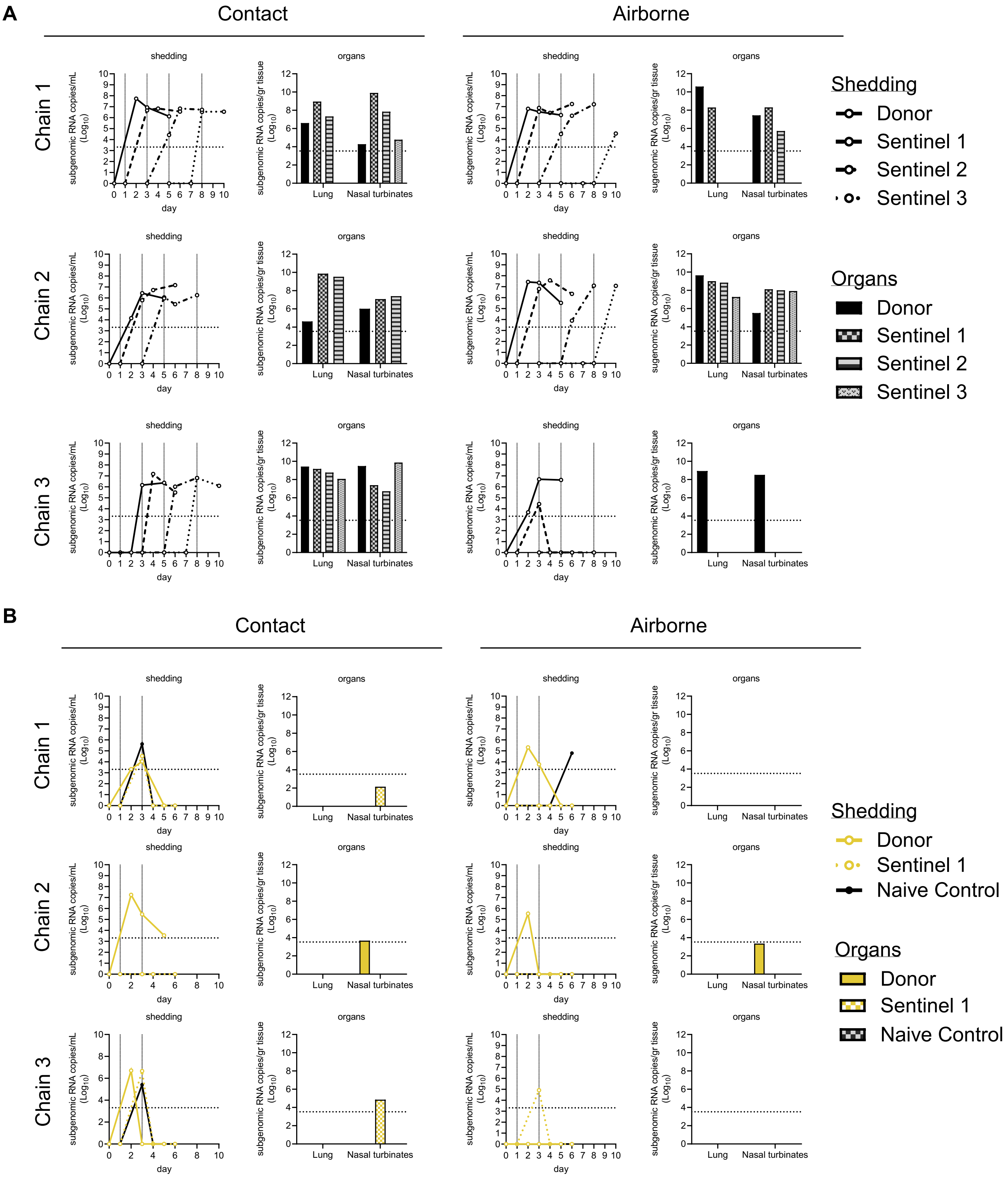
A
Contact
Airborne
Chain 1
Chain 2
Chain 3
shedding
organs
subgenomic RNA copies/mL (Log10)
subgenomic RNA copies/gr tissue (Log10)
day
Lung
Nasal turbinates
Shedding
Donor
Sentinel 1
Sentinel 2
Sentinel 3
Organs
Donor
Sentinel 1
Sentinel 2
Sentinel 3
B
Contact
Airborne
Chain 1
Chain 2
Chain 3
Shedding
Donor
Sentinel 1
Naive Control
Organs
Donor
Sentinel 1
Naive Control

C

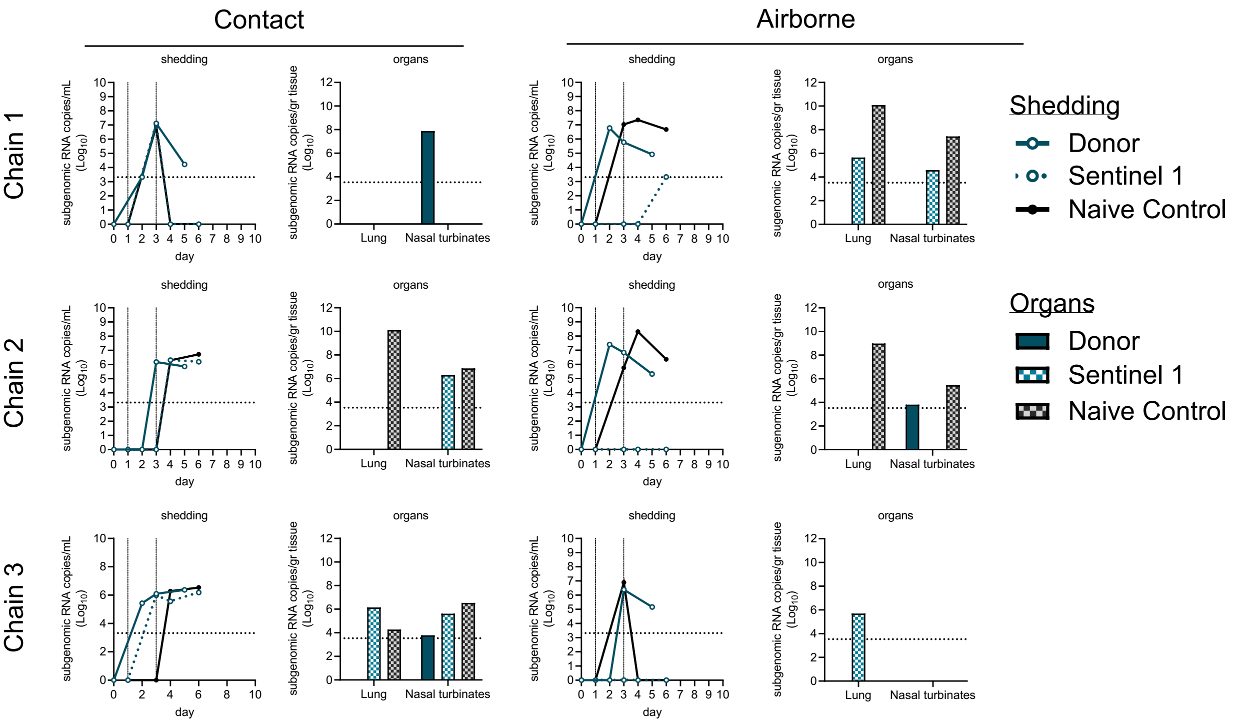

Contact
Airborne
Chain 1
Chain 2
Chain 3
shedding
organs
subgenomic RNA copies/mL (Log10)
subgenomic RNA copies/gr tissue (Log10)
day
Lung
Nasal turbinates
Shedding
Donor
Sentinel 1
Naive Control
Organs
Donor
Sentinel 1
Naive Control

D

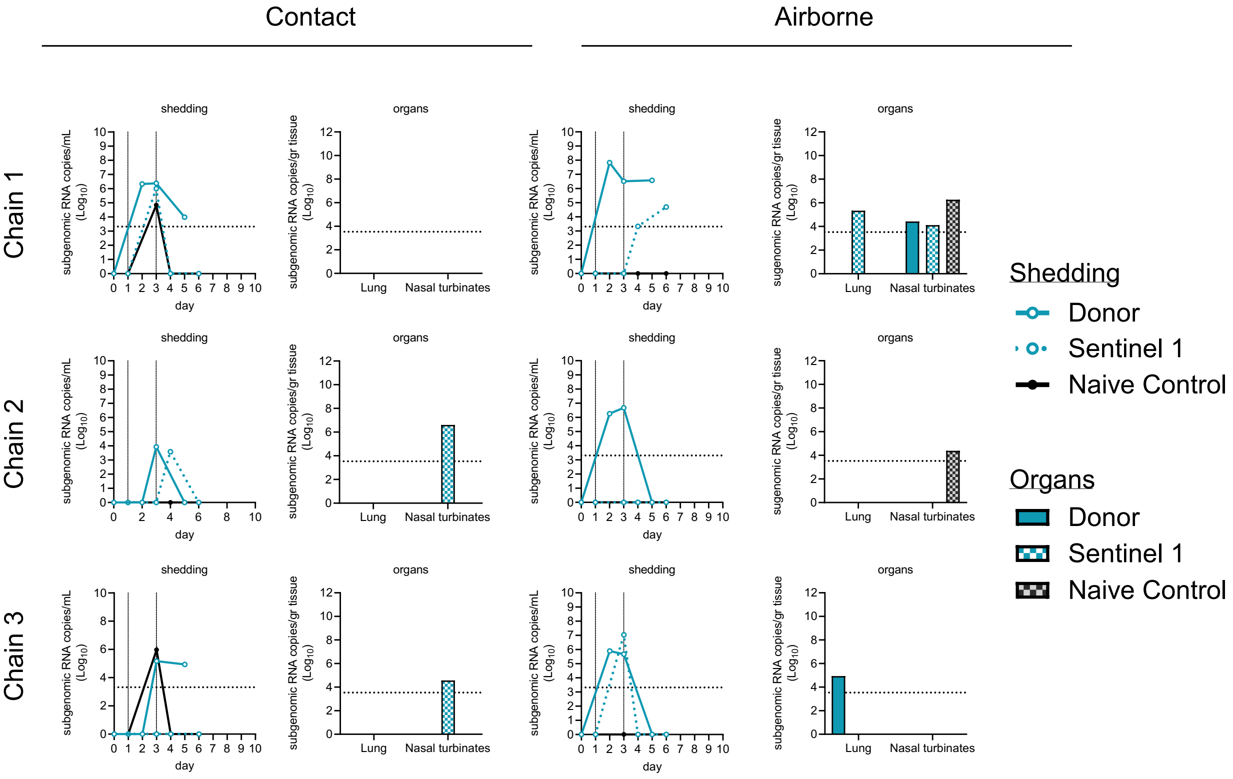

Contact
Airborne
Chain 1
Chain 2
Chain 3
shedding
organs
subgenomic RNA copies/mL (Log10)
subgenomic RNA copies/gr tissue (Log10)
day
Lung
Nasal turbinates
Shedding
Donor
Sentinel 1
Naive Control
Organs
Donor
Sentinel 1
Naive Control

Supplemental Figure 1. Shedding and organ titers of individual animals in each chain. Animals were grouped into either airborne or contact transmission chains by immune state (N = 3). Chains consisted of either a donor and three consecutive sentinels (naïve chains) or a donor and two sentinels, one with previous immunity and one naïve control (IM, IN, and PI chains). Donors were infected with 1 x 10^4 $TCID_{50}$ SARS-CoV-2 at a 1:1 ratio of Omicron and Delta and 24 hours later exposed to either a sentinel and naïve animal (IM, IN, and PI chains) or to the first sentinel (naïve chains) for 48 hours. Naïve chains continued in a similar fashion over four total animals. Replicating virus was measured via oropharyngeal swabs at 2, 3, and 5 DPI/DPE, and in lung and nasal turbinate samples at 5 DPI/DPE. Viral shedding is depicted in oropharyngeal swabs and viral replication in lung and nasal turbinate samples of all animals in contact (left) and airborne (right) chains. Viral shedding and replication are displayed as log_{10} (sgRNA copies/mL) in swabs and log_{10} (sgRNA copies/gram of tissue) in tissues. Horizontal lines indicate limit of detection. Vertical lines indicate exposure events in the chain. **A.** Naïve chains. **B.** Previously infected chains, **C.** Intramuscularly vaccinated chains. **D.** Intranasally vaccinated chains. Colours refer to legends on the right.

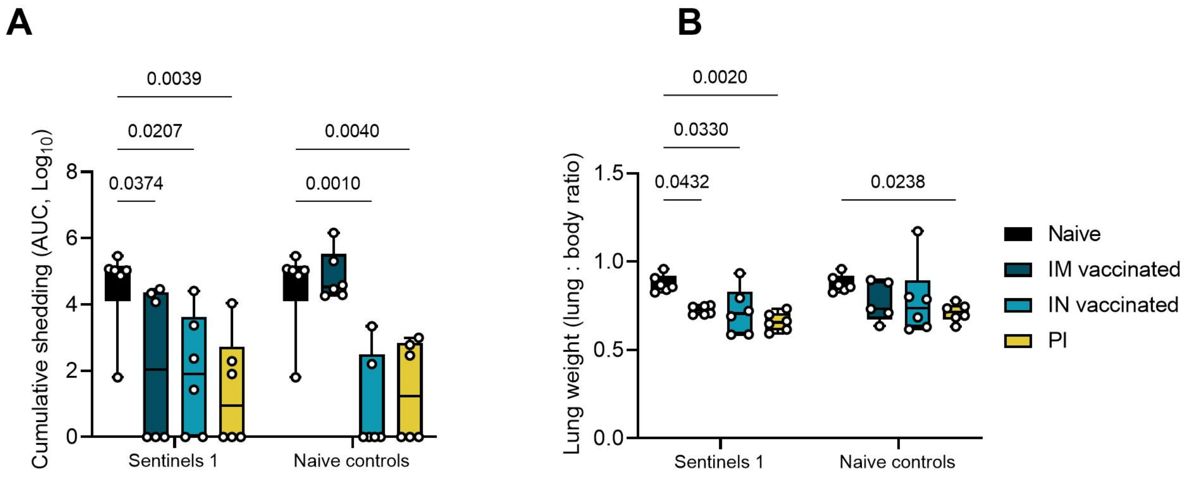

Supplemental Figure 2. Reduction of disease severity and shedding in sentinels. A. Cumulative shedding. Area under the curve (AUC) of sgRNA measured in oral swabs taken on 2, 3, and 5 DPE. Whisker-plots depicting median, min and max values, and individual values, N = 6, ordinary two-way ANOVA, followed by Šídák's multiple comparisons test. **B.** Lung samples were collected at 5 DPE for sentinels and naïve controls. Lung weights (lung:body weight ratio). Whisker-plots depicting median, min and max values, and individual values, N = 6, ordinary two-way ANOVA, followed by Šídák's multiple comparisons test. Black = naïve, dark blue = IM vaccinated, light blue = IN vaccinated, yellow = PI. P-values stated were significant (<0.05). Naïve sentinels 1 are depicted twice in each graph for visualization purposes only.

Supplemental Table 1: Pathological Assessment of IN, IM vaccinated or PI Syrian hamsters on day 5 post challenge. nsf = no significant findings. y = yes. n = no.

Group	Naïve						IM vaccinated						IN vaccinated						Previously infected					
Lesions visible grossly	y	y	n	y	y	y	y	y	y	?	y	n	n	n	n	N	n	n	n	n	?	n	n	n
Lung			nsf							nsf			nsf	nsf	nsf	nsf		nsf		nsf	nsf	nsf	nsf	
% total area affected	60	60		60	70	80	30	<10	20		20	<10					10		<10					<10
Interstitial Pneumonia																								
Syncytial Cell	y	y	n	y	y	y	y	n	n	n	n	n	n	n	n	n	n	n	n	n	n	n	n	n
Alveolar inflammation or exudate	y	y	n	y	y	y	y	y	y	n	y	y	n	n	n	n	n	n	n	n	n	n	n	n
Endotheleitis, vasculitis	y	y	n	y	y	y	y	y	y	n	y	n	n	n	n	n	n	n	n	n	n	n	n	n
Type II pneumocyte hyperplasia	n	n	n	n	n	n	y	n	n	n	n	n	n	n	n	n	n	n	n	n	n	n	n	n
Hemorrhage, fibrin and or edema	y	y	n	y	y	y	n	n	y	n	n	n	n	n	n	n	n	n	n	n	n	n	n	n
Bronchiolar hyperplasia	y	y	n	y	y	y	y	n	n	n	n	n	n	n	n	n	n	n	n	n	n	n	n	n
SARS-CoV-2 IHC bronchioles	3	2	0	2	2	2	0	0	3	0	0	1	0	0	0	0	0	0	0	0	0	0	0	0
SARS-CoV-2 IHC alveoli	4	3	0	3	4	4	1	0	3	0	0	0	0	0	0	0	0	0	0	0	0	0	0	0
Positive pixel analysis CD3	16	17	7	14	14	16	6	10	10	9	9	10	7	8	8	8	6	9	8	7	8	7	8	6
Positive pixel analysis PAX5	3	2	2	2	2	2	1	2	2	2	2	2	2	2	2	2	2	2	1	1	2	2	2	2

Supplemental Table 2: Shedding and tissue titers for each transmission chain. IM = intramuscularly vaccinated, IN = intranasally vaccinated, PI = Previously infected, BDL = Below qRT-PCR detection limit, sgRNA = sub-genomic RNA. Swab days = 1, 3 and 5 days

				sgRNA Ct-value					% Delta reads					% Omicron reads				
**Transmission route**	**Chain**	**Chain link**	**Immune status**	**Day 2 swab**	**Day 3 swab**	**Day 5 swab**	**Lung**	**Nasal turbinates**	**Day 2 swab**	**Day 3 swab**	**Day 5 swab**	**Lung**	**Nasal turbinates**	**Day 2 swab**	**Day 3 swab**	**Day 5 swab**	**Lung**	**Nasal turbinates**
air	1	Donor	IM	25	28	32	BDL	BDL	82	94	95			17	0	0		
air	1	Naïve Control	IM	24	23	26	15	23	99	100	100	100	100	0	0	0	0	0
air	1	Sentinel 1	IM	BDL	BDL	37	30	32					97					0
contact	1	Donor	IM	40	24	34	BDL	22		98	98		99	0	0	0		0
contact	1	Naïve Control	IM	25	BDL	BDL	BDL	BDL	94					0				
contact	1	Sentinel 1	IM	24	BDL	BDL	BDL	BDL	98					0				
air	2	Donor	IM	23	25	30	BDL	35	86	92	100		100	13	7	0		0
air	2	Naïve Control	IM	28	20	27	19	30	100	99	99	100	100	0	0	0	0	0
air	2	Sentinel 1	IM	BDL	BDL	BDL	BDL	BDL										
contact	2	Donor	IM	BDL	27	28	BDL	BDL		99	99				0	0	0	
contact	2	Naïve Control	IM	BDL	27	25	16	26		100	99	100	100		0	0	0	0
contact	2	Sentinel 1	IM	BDL	27	27	BDL	28		100	99		100		0	0		0
air	3	Donor	IM	BDL	26	31	BDL	BDL		99	99				0	0		
air	3	Naïve Control	IM	25	BDL	BDL	BDL	BDL	99					0	0			
air	3	Sentinel 1	IM	BDL	BDL	BDL	28	BDL										
contact	3	Donor	IM	30	27	27	BDL	35	94	99	100			0	0	0		0
contact	3	Naïve Control	IM	BDL	27	26	34	31		100	100		100		0	0		0
contact	3	Sentinel 1	IM	28	29	27	27	29	19	100	99	100	100	80	0	0	0	0
air	1	Donor	IN	21	26	26	BDL	33	81	92	98		81	18	7	0		
air	1	Naïve Control	IN	BDL	BDL	BDL	BDL	27					100					0
air	1	Sentinel 1	IN	BDL	BDL	BDL	BDL	BDL										
contact	1	Donor	IN	27	26	35	BDL	BDL	98	95	98			0	0	0		

contact	1	Naïve Control	IN	32	BDL	BDL	BDL	BDL	100					0				
contact	1	Sentinel 1	IN	28	BDL	BDL	BDL	BDL	100					0				
air	2	Donor	IN	26	25	BDL	BDL	BDL	84	93				15	0			
air	2	Naïve Control	IN	BDL	BDL	BDL	BDL	33					100					0
air	2	Sentinel 1	IN	BDL	BDL	BDL	BDL	BDL										
contact	2	Donor	IN	BDL	35	BDL	BDL	BDL		76					23			
contact	2	Naïve Control	IN	BDL	BDL	BDL	BDL	BDL										
contact	2	Sentinel 1	IN	BDL	36	BDL	BDL	27		100					0			
air	3	Donor	IN	28	29	BDL	31	BDL	63	84				36	15			
air	3	Naïve Control	IN	BDL	BDL	BDL	BDL	BDL										
air	3	Sentinel 1	IN	24	BDL	BDL	BDL	BDL	75					24				
contact	3	Donor	IN	BDL	31	32	BDL	BDL		99	100				0	0		
contact	3	Naïve Control	IN	28	BDL	BDL	BDL	BDL	97					0				
contact	3	Sentinel 1	IN	BDL	BDL	BDL	BDL	32					100					0
air	1	Donor	Naïve	25	26	27	14	23	83	92	99	100	99	16	7	0	0	0
air	1	Sentinel 1	Naïve	24	26	24	20	21	100	90	84	100	99	0	9	15	0	0
air	1	Sentinel 2	Naïve	32	27	24	BDL	29	100	99	100		100	0	0	0		0
air	1	Sentinel 3	Naïve	BDL	BDL	33	BDL	BDL			100					0		
contact	1	Donor	Naïve	22	25	27	25	33	93	98	98	100	97	0	0	0	0	0
contact	1	Sentinel 1	Naïve	25	25	26	18	16	96	99	99	99	100	0	0	0	0	0
contact	1	Sentinel 2	Naïve	33	25	25	23	21	83	100	99		100	16	0	0		0
contact	1	Sentinel 3	Naïve	BDL	26	26	BDL	32		100	100		100		0	0		0
air	2	Donor	Naïve	23	23	30	16	29	82	85	99	99	98	17	14	0	0	0
air	2	Sentinel 1	Naïve	25	22	27	19	22	87	100	100	99	100	12	0	0	0	0
air	2	Sentinel 2	Naïve	BDL	34	24	20	23		100	100	100	100		0	0	0	0
air	2	Sentinel 3	Naïve	BDL	BDL	24	24	22			99		100		0	0		0
contact	2	Donor	Naïve	34	26	28	32	27	100	97	97	100	99	0	0	0	0	0

contact	2	Sentinel 1	Naïve	29	25	24	15	24	100	99	99	100	99	0	0	0	0	0
contact	2	Sentinel 2	Naïve	28	30	27	17	23	100	100	99	100	100	0	0	0	0	0
air	3	Donor	Naïve	36	25	26	19	20	100	99	99	100	99	0	0	0	0	0
air	3	Sentinel 1	Naïve	33	BDL	BDL	BDL	BDL	100					0				
air	3	Sentinel 2	Naïve	BDL	BDL	BDL	BDL	BDL										
contact	3	Donor	Naïve	BDL	25	28	21	16		100	99	100	100		0	0	0	0
contact	3	Sentinel 1	Naïve	BDL	27	27	17	17		99	99	99			0	0	0	
contact	3	Sentinel 2	Naïve	BDL	24	30	18	25		97	99	100	100		0	0	0	0
contact	3	Sentinel 3	Naïve	BDL	28	25	20	27		100	99	100	100		0	0	0	0
air	1	Donor	PI	32	35	BDL	BDL	BDL	0	0				93	95			
air	1	Naïve Control	PI	BDL	BDL	32	BDL	BDL			100					0		
air	1	Sentinel 1	PI	BDL	BDL	BDL	BDL	BDL										
contact	1	Donor	PI	37	35	BDL	BDL	BDL		100				0	0			
contact	1	Naïve Control	PI	29	BDL	BDL	BDL	BDL										
contact	1	Sentinel 1	PI	33	BDL	BDL	BDL	39	86					13				0
air	2	Donor	PI	29	BDL	BDL	BDL	37	0					95	99			0
air	2	Naïve Control	PI	BDL	BDL	BDL	BDL	BDL										
air	2	Sentinel 1	PI	BDL	BDL	BDL	BDL	BDL										
contact	2	Donor	PI	23	30	37	BDL	35	95	96	57		95	0	0	42		0
contact	2	Naïve Control	PI	BDL	BDL	BDL	BDL	BDL										
contact	2	Sentinel 1	PI	BDL	BDL	BDL	BDL	BDL										
air	3	Donor	PI	BDL	BDL	BDL	BDL	BDL										
air	3	Naïve Control	PI	BDL	BDL	BDL	BDL	BDL										
air	3	Sentinel 1	PI	32	BDL	BDL	BDL	BDL	91					8				
contact	3	Donor	PI	25	BDL	BDL	BDL	BDL	100					0				
contact	3	Naïve Control	PI	30	BDL	BDL	BDL	BDL	100					0				
contact	3	Sentinel 1	PI	25	BDL	BDL	BDL	32	100					0				

Supplemental Table 3: SARS-CoV-2 isolates used in this study.

Virus	WHO Description	PANGO Lineage	GiSAID/Genbank Acc
SARS-CoV-2/human/USA/WA-CDC-WA1/2020	Lineage A	WA1	MN985325
England/204820464/2020	Alpha	B.1.1.7	EPI_ISL_683466
USA/MD-HP01542/2021	Beta	B.1.351	EPI_ISL_890360
hCoV-19/USA/MD-HP03867/2021	Gamma	P.1	EPI_ISL_1468644
hCoV-19/USA/KY-CDC-2-4242084/2021	Delta	B.1.617.2	EPI_ISL_1823618
hCoV-19/USA/GA-EHC-2811C/2021	Omicron	B.1.1.529	EPI_ISL_7171744

Supplemental Table 4: Pathological Assessment of IN and IT inoculated Syrian hamsters on day 5. nsf = no significant findings. y = yes. n = no.

**Group**	**IN**						**IT**					
lesions visible grossly	n	n	n	y	n	n	y	y	y	y	y	y
Percentage of total area affected		<5		10	5		50	90	70	50	70	60
Alveolar inflammation or exudate	n	n	1	2	1	1	4	4	4	4	4	4
Bronchiolar epithelial cell inflammation/necrosis	n	1	1	n	n	n	1	n	n	n	n	n
Endotheleitis, vasculitis	n	n	n	y	n	n	y	y	y	y	y	y
Hemorrhage, fibrin and or edema	n	n	n	n	n	n	n	3	3	n	2	n
SARS2 IHC bronchioles	0	1	1	1	1	1	0	1	0	0	0	0
SARS2 IHC alveoli	0	1	1	2	1	1	4	4	4	4	4	4
